# Complete Genome Sequence of Emerging Porcine Circovirus Types 2a and 2b from India

**DOI:** 10.1128/genomeA.00087-15

**Published:** 2015-03-12

**Authors:** Uttaran Bhattacharjee, Anuj Ahuja, Indu Sharma, Amarjit Karam, Amit Kumar Chakraborty, Sandeep Ghatak, Kekungu Puro, Samir Das, Ingudam Shakuntala, Sarbani Giri, R. K. Pegu, R. Laha, A. Pattanayak, S. V. Ngachan, Arnab Sen

**Affiliations:** a Division of Animal Health, ICAR RC for NEH Region, Umiam, Meghalaya, India; b Department of Microbiology, School of Life Sciences, Assam University, Silchar, India; c Department of Life Science and Bio-informatics, School of Life Sciences, Assam University, Silchar, India

## Abstract

We report here the first characterized complete genome sequence of porcine circovirus types 2a and 2b from northeastern states of India. These isolates may serve as a potential reference for the Indian strains of porcine circovirus types 2a and 2b.

## GENOME ANNOUNCEMENT

Porcine circovirus-2 (PCV-2), a member of the *Circovirade* family, is a small, nonenveloped single stranded circular DNA virus. PCV-2 is considered an important emerging pathogen for global swine industries with several disease manifestations that are collectively called PCV-2-associated disease (PCVAD) and include post weaning multisystemic wasting syndrome (PMWS) (1). PMWS was first reported in 1997 in Canada and subsequently from other countries including India (2, 3).

The circular genome (1,767 to 1,768 bp) of PCV-2 is comprised of three major open reading frames (ORFs), i.e., ORF-1 which encodes the replication protein (rep and rep[2032]), ORF-2 considered as a phylogenetic marker and encodes the capsid protein (cap), and ORF-3 which encodes the apoptotic protein (4). PCV-2 is subdivided into three genotypes based on their ORF-2 gene, PCV-2a, PCV-2b, and PCV-2c (5, 6).

The present study was conducted at organized farms of two states in northeastern India (Arunachal Pradesh and Meghalaya) with histories of reproductive and respiratory disorders. In northeastern India, pig husbandry plays an integral socio-economic role and the region is home to about 40% of the total pig population of India (7). The PCV-2 genomes of AR-1 (Arunachal Pradesh) and ML-7 (Meghalaya) were amplified with previously reported primers (8). The PCR products were purified, cloned into pGEMT, and sequenced using BigDye technology on an ABI 3500xL Genetic Analyzer (Applied Biosystems). Following assembly, the PCV-2 genome was 1,768 to 1,767 bp long and encoded three major ORFs of PCV-2. To determine the genotypes of the isolates, 100 sequences of PCV-2 were downloaded from GenBank (http://www.ncbi.nlm.nih.gov/Genbank), comprising the classical prototypes of genotypes PCV-2a (accession no. AF055392), PCV-2b (accession no. AF055394), and PCV-2c (accession no. EU148503) and were aligned using Clustal Omega (http://www.ebi.ac.uk/Tools/msa/clustalo/). A phylogenetic neighbor-joining tree based on ORF-2 of these isolates was built using the p-distance method calculated with 1,000 bootstrap replicates in MEGA 5.0. Phylogenetic analysis suggested that AR-1 and ML-7 isolates are closely related to PCV-2a and PCV-2b with 98% and 99.9% similarity, respectively.

The length of the replicase gene (ORF-1) was 945 bp and it encodes 315 amino acids in the replication protein. In the case of the capsid gene (ORF-2), the length was 702 bp in AR-1 and 705 bp in ML-7 and 234 to 235 amino acids are encoded in the capsid protein, respectively. The predicted amino acid sequence of the ORF-2s of AR-1 and ML-7 showed high levels of identity to PCV-2a strains (95.7% to 97%) and PCV-2b strains (93.6% to 94.4%), respectively.

This is the first report regarding whole-genome communication of PCV-2a and PCV-2b from India and these isolates could help us to better understand the prevalent genogroups of PCV-2 in India.

### Nucleotide sequence accession numbers.

The complete genome sequences of PCV-2 isolates AR-1 and ML-7 have been deposited in DDBJ under accession no. LC004732 and LC004742, respectively.
